# Role of Fos-related antigen 1 in the progression and prognosis of ductal breast carcinoma

**DOI:** 10.1111/j.1365-2559.2011.03785.x

**Published:** 2011-03

**Authors:** Angela Flavia Logullo, Mônica Maria Ágata Stiepcich, Cintia Aparecida Bueno de Toledo Osório, Sueli Nonogaki, Fátima Solange Pasini, Rafael Malagoli Rocha, Fernando Augusto Soares, Maria M Brentani

**Affiliations:** Departamento de Patologia, UNIFESPSão Paulo, Brazil; 1 Departamento de Patologia, Hospital A. C. CamargoSão Paulo, Brazil; 2 Departamento de Patologia, Instituto Adolfo LutzSão Paulo, Brazil; 3 Disciplina de Oncologia (LIM24), Departamento de Radiologia e Oncologia, da Faculdade de Medicina da USPSão Paulo, Brazil

**Keywords:** ductal breast carcinoma, Fra-1, prognosis, progression

## Abstract

**Aims:**

Fos-related antigen 1 (Fra-1) is a member of the activator protein 1 (AP-1) transcription factor family. Our objective was to evaluate the role of Fra-1 expression in breast carcinoma progression and prognosis.

**Methods and results:**

Fra-1 expression was investigated by immunohistochemistry in two tissue microarrays containing, respectively, 85 ductal carcinoma *in situ* (DCIS) and 771 invasive ductal carcinoma (IDC) samples. Staining was observed in the nucleus and cytoplasm of the carcinomas, but only nuclear staining was considered to be positive. Fibroblasts associated with IDC were also Fra-1-positive. The frequency of Fra-1 positivity in IDC (22.8%) was lower than that in DCIS (42.2%). No association was found between Fra-1 and clinico-pathological variables in DCIS. In IDC, Fra-1 expression correlated with aggressive phenotype markers, including: high grade, oestrogen receptor negativity and human epidermal growth factor receptor 2 (HER-2) positivity (*P* = 0.001, 0.015 and 0.004, respectively), and marginally with the presence of metastasis (*P* = 0.07). Fra-1 was more frequently positive in basal-like (34%) and in HER-2-positive (38.5%) subtypes than in luminal subtypes. Fra-1 presence did not correlate with survival.

**Conclusions:**

A high frequency of Fra-1 in DCIS tumours may be associated with early events in breast carcinogenesis. Although Fra-1 expression correlated with features of a more aggressive phenotype in IDC, no relationship with overall survival was found.

## Introduction

Progression of breast cancer is often accompanied by changes in the pattern of gene expression of neoplastic cells, resulting in a highly tumorigenic and invasive cell phenotype. All of these properties are hypothesized to depend on deregulated transcription.^1^

The transcription activator protein, activator protein 1 (AP-1), has been broadly implicated in tumour progression, and many genes involved in cell proliferation, differentiation, malignant transformation and cell invasion are AP-1-dependent.^2^ Fos-related antigen 1 (Fra-1) is a member of the Fos protein family (c-Fos, Fos B, Fra-1 and Fos-related antigen 2), which are able to dimerize with members of the Jun family, forming AP-1. There are several lines of evidence indicating that Fra-1 is associated with a more malignant phenotype and could play a pivotal role in cancer progression.^3^ An association between Fra-1 and mesenchymal characteristics in highly invasive breast cancer cells was reported, suggesting that Fra-1 might be a component of the molecular switch in the epithelial–mesenchymal transition (EMT), a programme by which epithelial cells acquire motility, invasiveness and resistance to apoptosis.^4^–^10^ A potential role of Fra-1 in the regulation of the expression of vimentin, a key molecule of EMT, and a requirement for Fra-1 in motility have been suggested.^7^,^11^

The majority of studies concerning the role of Fra-1 in breast cancer have focused on cell culture systems.^4^–^12^ Indeed, there are few reports on the expression of Fra-1 in human breast carcinoma samples.^13^–^15^ Thus, in the present work, we attempted to explore the role of Fra-1 in the progression and prognosis of breast cancer, by undertaking a retrospective immunohistochemical analysis of Fra-1 expression in a series of ductal carcinoma *in situ* (DCIS) and invasive ductal carcinoma (IDC) samples within tissue microarrays (TMAs), and performing a comparative analysis of Fra-1 expression prognostic value with classical prognostic markers and patient outcome.

## Materials and methods

### Tumour samples and clinical data

Formalin-fixed and paraffin-embedded tissue specimens from patients with IDC and DCIS diagnosed at the A. C. Camargo Cancer Hospital (São Paulo, Brazil) were included in this study after approval by the institutional review board. Because the study was retrospective, informed patient consent was not required. A TMA containing 771 samples of primary IDC diagnosed from 1980 to 2005, and an additional TMA containing 85 samples of DCIS lesions [45 associated with an invasive carcinoma component and 40 without an invasive component (pure DCIS)] diagnosed from 1980 to 2001, were produced. The IDC cases studied constituted an independent cohort and were not paired with the DCIS/IDC cases analysed. All cases were reviewed by C.T.O., M.S. and F.A.S. to corroborate the diagnosis. The characteristics of these two retrospective cohorts are given in Table 1. Patients were enrolled according to the inclusion criteria, consisting of suitable paraffin blocks for immunohistochemistry, adequate clinical parameters and follow-up information. The Nottingham system was used for assessment of histological grade of the invasive cases.^16^ Nuclear grade, based on the consensus Conference Committee Anonymous,^17^ was used to classify DCIS cases. In all IDC cases, the treatment involved mastectomy, radiotherapy and axillary lymph node dissection. Cases with a positive immunohistochemical oestrogen receptor (ER) result received hormone therapy, the others were treated with chemotherapy. The median follow-up of both cohorts (IDC and DCIS) was 70 months. At the final follow-up (July 2007), 367 IDC patients were alive and 404 had died of disease. At the final follow-up of the DCIS patients (February 2008), 60 patients were alive and 12 had died. None of the patients with pure DCIS had experienced recurrence or progression to an invasive cancer within the median follow-up time.

**Table 1:** Clinicopathological variables in ductal breast carcinoma patients

Characteristics	*In situ* (*n* = 85), no. (%)	Invasive (*n* = 771), no. (%)
Median age, years (range)	50.5 (20–83)	56 (33–80)

Histology		
Comedo	6 (7.1%)	Not aplicable

Not comedo	79 (92.9%)	Not aplicable

Nodal status		
N0	Not aplicable	233 (27.9%)

N+	Not aplicable	533 (62.3%)

Not known	Not aplicable	5 (0.6%)

Tumor size		
T1 + T2		339 (44.0%)

T3 + T4		432 (56.0%)

Disease stage		
TxN0M0	Not aplicable	226 (29.3%)

TxN1M0	Not aplicable	221 (28.7%)

TxN2-3M0	Not aplicable	252 (32.7%)

TxNxM1	Not aplicable	72 (9.3%)

Histological grade		
1	11 (12.9%)	99 (12.9%)

2	38 (44.7%)	444 (57.7%)

3	34 (40.0%)	226 (29.4%)

Not known	2 (2.4%)	2 (0.3%)

Status		
Alive	60 (83.3%)	367 (47.6%)

Deceased	12 (16.7%)	404 (52.4%)

Oestrogen receptors		
Positive	58 (68.2%)	500 (64.9%)

Negative	24 (28.2%)	266 (34.5%)

Not known	3 (3.5%)	5 (0.6%)

Progesterone receptors		
Positive	48 (56.5%)	351 (45.5%)

Negative	34 (40.0%)	409 (53.0%)

Not known	3 (3.5%)	11 (1.4%)

HER-2		
Positive	34 (40.0%)	105 (13.6%)

Negative	45 (52.9%)	650 (84.3%)

Not known	6 (7.1%)	16 (2.1%)

HER-2, Human epidermal growth factor receptor 2.

### TMA construction

The procedure for construction of TMAs was as previously described.^18^ Briefly, cylinders 1 mm in diameter were punched from selected areas of the donor paraffin blocks (Beecher Instruments, Silver Spring, MD, USA). Each case was sampled twice and distributed in four new blocks, which were stored at 4°C, before sections 4 μm thick were prepared for each marker to be examined by immunohistochemistry. Normal breast tissue corresponding to a macroscopically healthy region distinct from neoplastic lesions were utilized as controls (*n* = 4).

### Immunohistochemistry

Monoclonal antibodies against cytokeratin (CK) 5/6 (clone D5/16B4), progesterone receptor (PR) (clone PgR636) and human epidermal growth factor receptor 2 (HER-2) (polyclonal) were obtained from Dako (Glostrup, Denmark) and diluted 1:100, 1:200 and 1:1000, respectively. Fra-1 (C12 SC-28310) monoclonal antibody raised against amino acids 1–50 of human Fra-1 was purchased from Santa Cruz Biotechnology (Santa Cruz, CA, USA) and diluted 1:100. Each slide was also stained with anti-ER (clone 6F-11, 1:50; Neomarkers, Fremont, CA, USA) and anti-CK14 (clone LL02, 1:400; BioGenex, Fremont, CA, USA). We performed optimization, in order to standardise the immunohistochemical staining for the Fra-1 primary antibody, concerning antigen retrieval method (equipment for humid heat, pH, and type of buffer), dilution of primary antibody, and the visualization system method. After deparaffinisation and rehydration of the TMA sections, antigen retrieval was performed in a pressure cooker. After primary antibody incubation and a polymer–peroxidase (Novocastra, Newcastle, UK) amplification step, antigen detection was carried out in a solution containing 3,3-Diaminobenzidine (Sigma, St Louis, MO, USA) and 6% H_2_O_2_. Counterstaining was performed with Harris haematoxylin. Positive controls were included in each staining reaction, and consisted of breast cancers known to express each of the antigens of interest. The Fra-1-positive control was a case of cervical squamous epithelium. An IDC sample was also used as a positive control. Omission of primary antibody was used as a negative control in the same sample. Normal breast could also be interpreted as an internal negative control in the samples. Specimens that exhibited a complete absence of staining or ≤10% of positive cells were considered to be Fra-1-negative.

An Allred score of ER and PR immunoreactivity ≤2 was considered to be negative result.^19^ For HER-2 samples, lack of reactivity in <10% of the tumour cells was scored as zero. Barely perceptible focal membrane staining was scored as one. Weak to moderate staining observed in >10% of the tumour cells was scored as two. Strong complete membrane staining in >10% of the tumour cells was scored as three. We considered the result to be positive only if the score was 3+, according to American Society of Clinical Oncology of American Pathologists recommendations.^20^ For basal CKs (CK5/6 and CK14), the sample was considered to be positive if ≥10% of the tumour cells were reactive. Samples with immunoreactivity for at least one of the basal CKs (CK5/6 or CK14) was considered to be CK-positive.

### Statistical methods

Correlations between antigen expression and other clinico-pathological parameters were studied with the chi-square test. Survival probabilities were estimated by the univariate Kaplan–Meier method and survival curves were compared with the log rank test (Mantel–Haenszel method). ssps version 10.0 for Windows (SPSS Inc., Chicago, IL, USA) was used for analyses.

## Results

**Figure 1:**
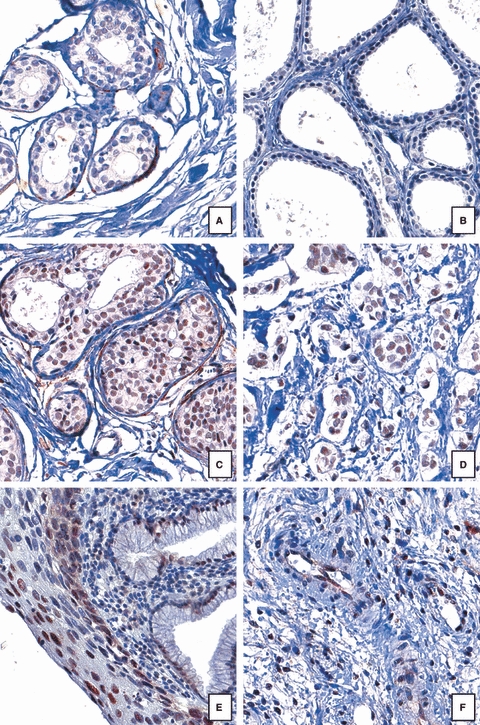
Fos-related antigen 1 (Fra-1) stained by immunohistochemistry in different sections observed at ×400 magnification. **A**, negative normal breast. **B**, negative breast adenosis. **C**, positive ductal carcinoma *in situ*. **D**, strongly positive invasive ductal carcinoma. **E**, positive control of cervix and clear negative stroma. **F**, positive endothelia and fibroblasts in contrast to clear negative stroma.

The pattern of Fra-1 expression in IDC was purely nuclear or mixed nuclear and cytoplasmic, but the reactivity in the latter component was weaker than that of nuclear staining. Reactivity was scored as positive or negative according to the frequency of labelled nuclei of the tumour cells. We reasoned that nuclear localization of Fra-1 is important for its function as a transcription factor. The pattern of Fra-1 expression and localization in DCIS was similar to that in IDC. Fra-1 immunoreactivity was not present in normal breast tissues or in the breast adenosis cases used as controls. In Figure 1, we show some representative immunohistochemical reactivity. We obtained reliable immunohistochemical staining, with a sharp pattern of nuclear staining and a clear background with no non-specific stromal or parenchymal staining.

Pure DCIS (*n* = 40) and DCIS associated with an invasive component (*n* = 45) cases were similar with respect to nuclear grade (non-high grade 57.9% versus 60%, respectively, *P* = 1.0), ER positivity (71.8% versus 64.1%, *P* = 0.63), and PR positivity (61.5% versus 53.8%, *P* = 0.65). However, HER-2 frequency was statistically lower in the DCIS with IDC than in the pure DCIS cases (30.0% versus 56.4%, *P* = 0.024).

Table 2 summarizes Fra-1 protein expression in DCIS and IDC. Fra-1 staining was positive in 22.8% of IDC cases (176/771), and this percentage was significantly lower than that observed in DCIS (42.2%, *P* < 0.001). Differences in the proportion of Fra-1 positivity between pure DCIS and those DCIS lesions associated with an invasive component were not observed. A significantly higher frequency of HER-2-positive expression in DCIS than in IDC was also verified (*P* < 0.001).

**Table 2:** Comparison of Fos-related antigen 1 (Fra-1) and human epidermal growth factor receptor 2 (HER-2) protein expression between *in situ* and invasive ductal breast carcinoma

	Fra-1 expression			HER-2 expression		
		
	Negative, no. (%)	Positive, no. (%)	*P*-value	Negative, no. (%)	Positive, no. (%)	*P*-value
DCIS						

Pure	23 (57.5)	17 (42.5)		17 (43.6)	22 (56.4)	

With invasive component	26 (57.8)	19 (42.2)	<0.001	28 (70.0)	12 (30.0)	<0.001

IDC	595 (77.2)	176 (22.8)		650 (86.1)	105 (13.9)	

DCIS, Ductal carcinoma *in situ*; IDC, invasive ductal carcinoma.

Fra-1 expression: negative = no expression to +; positive = ++ to +++. HER-2 expression: negative = no expression to ++; positive = +++.

*P* = Statistical significance by chi-square test.

The relationships between Fra-1 expression and clinico-pathological features in DCIS are shown in Table 3. No significant associations were found between Fra-1 expression and pathological variables such as nuclear grade, presence of ER or PR and HER-2 positivity in the DCIS cases.

**Table 3:** Correlation of Fos-related antigen 1 (Fra-1) expression with prognostic factors in ductal carcinoma *in situ*

	Fra-1 expression		
		
Characteristics	Negative, no. (%)	Positive, no. (%)	*P*-value
Nuclear grade			
1	6 (54.5)	5 (45.5)	0.66
	
2	24 (63.2)	14 (36.8)	
	
3	18 (52.9)	16 (47.1)	

Oestrogen receptors			
Positive	30 (56.6)	23 (43.4)	0.62
	
Negative	16 (64.0)	9 (36.0)	

Progesterone receptors			
Positive	24 (53.3)	21 (46.7)	0.25
	
Negative	22 (66.7)	11 (33.3)	

HER-2			
Positive	18 (52.9)	16 (47.1)	0.49
	
Negative	28 (62.2	17 (37.8)	

HER-2, Human epidermal growth factor receptor 2.

*P* = Statistical significance by chi-square test. A two-sided *P* < 0.05 was considered to be statistically significant.

In contrast, a comparison between Fra-1 expression and clinico-pathological variables in IDC (Table 4) revealed an association between Fra-1 positivity and histological grade 2 or 3 tumours (*P* = 0.001), lack of ER expression (*P* = 0.015) and the presence of HER-2 overexpression (*P* < 0.004).

**Table 4:** Summary of the relationship between Fos-related antigen 1 (Fra-1) protein expression and clinico-pathological features in invasive ductal breast carcinoma

	Fra-1 expression		
		
Characteristics	Negative, no. (%)	Positive, no. (%)	*P*-value
Nodal status			
N0	179 (76.8)	54 (23.2)	0.852
	
N+	413 (77.5)	120 (22.5)	

Tumour size			
T1 + T2	257 (75.8)	82 (24.2)	0.438
	
T3 + T4	338 (78.2)	94 (21.8)	

Metastases status			
M0	493 (76.0)	156 (24.0)	0.077
	
M1	102 (83.6)	20 (16.4)	

Histological grade			
1	87 (87.9)	12 (12.1)	0.001
	
2	349 (78.6)	95 (21.4)	
	
3	158 (69.9)	68 (30.1)	

Oestrogen receptors			
Positive	399 (79.8)	101 (20.2)	0.015
	
Negative	191 (71.8)	75 (28.2)	

Progesterone receptors			
Positive	274 (78.1)	77 (21.9)	0.604
	
Negative	312 (76.3)	97 (23.7)	

HER-2			
Positive	69 (65.7)	36 (34.3)	0.004
	
Negative	514 (79.1)	136 (20.9)	

HER-2, Human epidermal growth factor receptor 2.

*P* = Statistical significance by chi-square test. A two-sided *P* < 0.05 was considered to be statistically significant.

Considering the molecular profiles, as previously defined by Rakha *et al.*,^21^ we classified breast carcinoma samples into subgroups. Lesions were characterized as luminal A (HER-2-negative/ER-positive; 452 cases), luminal B (HER-2-positive/ER-positive; 38 cases), HER-2 rich (HER-2-positive/ER-negative/PR-negative; 65 cases), basal-like (HER-2-negative/ER-negative/PR-negative/CK5/6-positive and/or CK14-positive; 47 cases) and triple-negative (HER2-negative/ER-negative/PR-negative but without expression of CK5/6 and CK14; 134 cases). The frequency of Fra-1 positivity was significantly different among these subgroups (*P* = 0.002): 19% in luminal A cases, 28.9% in luminal B cases, 34% in basal-like cases and 20.9% in triple-negative cases. The HER-2-positive group had the highest proportion of tumours positive for Fra-1 (38.5%) (Table 5).

**Table 5:** Distribution of Fos-related antigen 1 (Fra-1) protein frequency in subtypes of invasive ductal breast carcinoma

Subtype	Total no. of patients	Positive Fra-1 frequency, no. (%)	*P*-value
Luminal A	452	86 (19.0)	0.002
	
Luminal B	38	11 (28.9)	
	
Basal-like	47	16 (34.0)	
	
Triple-negative/CK-negative	134	28 (20.9)	
	
HER-2	65	25 (38.5)	

CK, Cytokeratin; ER, oestrogen receptor; HER-2, human epidermal growth factor receptor 2; PR, progesterone receptor.

Luminal A: tumours HER-2-negative/ER-positive. Luminal B: tumours HER-2-positive/ER-positive. Basal-like: triple-negative and CK5/6-positive or CK14-positive. Triple-negative/CK-negative: HER-2/ER/PR-negative and CK5/6-negative and CK14-negative. HER-2: tumours HER-2-positive/ER-negative and PR-negative.

*P* = Statistical significance by chi-square test. A two-sided *P* < 0.05 was considered to be statistically significant.

To determine whether Fra-1 expression was related to patient survival, we prepared Kaplan–Meier survival curves and analysed them statistically (log rank test). Because several clinical parameters are known to affect survival, we also analysed prognostic indicators. Survival was found to be reduced in patients with compromised lymph nodes (*P* < 0.001), with ER-negative/PR-negative tumours or with large tumours (T_3_ + T_4_) (*P* < 0.001 for both). When patient survival was stratified by histological grade, differences in survival were noted between grades (1, 2 and 3, *P* = 0.001). Patients with grade 2 (*P* = 0.02) or grade 3 (*P* = 0.001) tumours had worse survival than those with grade 1 tumours. Survival curves showed a poorer outcome for the HER-2 and triple-negative subgroups (median survival: 4 and 5 years, respectively) and the basal-like subgroup (8 years) compared to luminal A (11 years) or luminal B cases (13 years) (*P* = 0.002). When all patients were considered, no differences in terms of survival were observed between patients with tumours scored as Fra-1-positive and those with low or negative levels (*P* = 0.66) (Table 6).

**Table 6:** Survival according to pathological and immunohistochemical parameters in invasive ductal breast carcinoma by univariate analysis

	No. of patients	Median survival (years)	95% CI	*P*-value
Patient subgroup				
Luminal A	452	11.0	8.66–13.33	0.002
	
Luminal B	38	13.0	1.18–24.82	
	
Basal-like	47	8.0	1.70–14.29	
	
Triple-negative/CK-negative	134	5.0	3.34–6.65	
	
HER-2	65	4.0	0.00–9.69	

Nodal status				
N0	233	Not reached	–	<0.001
	
N+	533	6.0	5.02–6.97	

Tumour size				
T1 + T2	339	Not reached	–	<0.001
	
T3 + T4	432	4.0	3.37–4.63	

Histological grade				
1	99	14.0	–	0.001
	
2	444	9.0	7.13–10.86	
	
3	226	7.0	4.96–9.03	

Fra-1 expression				
Negative	595	8.0	6.36–9.63	0.66
	
Positive	176	8.0	4.53–11.46	

CI, Confidence interval; CK, cytokeratin; ER, oestrogen receptor; Fra-1, Fos-related antigen 1; HER-2, human epidermal growth factor receptor 2; PR, progesterone receptor.

Luminal A: tumours HER2-negative/ER-positive. Luminal B: tumours HER2-positive/ER-positive. Basal-like: triple-negative and CK5/6-positive or CK14-positive. Triple-negative/CK-negative: HER-2/ER/PR-negative and CK5/6-negative and CK14-negative. HER-2: tumours HER-2-positive/ER-negative and PR-negative.

*P* = Statistical significance by log rank test.

## Discussion

We analysed Fra-1 expression in more than 800 breast cancers arranged in a TMA. Samples comprised pure DCIS cases, cases of DCIS within an invasive carcinoma, and more than 700 cases of IDC. This scenario enabled us to determine if Fra-1 expression changed during breast cancer progression. Fra-1 expression was identified within the nucleus but in some cases, cytoplasmic reactivity was also seen; the latter was disregarded for analysis. Other studies have previously described this pattern, but with the use of different antibodies.^13^,^14^

Our results obtained with pure DCIS and invasive carcinomas show that the frequency of Fra-1 expression in infiltrative lesions was significantly lower than in DCIS. Both previous studies investigating Fra-1 by immunohistochemistry in IDC and DCIS samples found Fra-1-positive staining in all analysed cases, in disagreement with our data.^13^,^14^ The reason for such differences is uncertain, but may be attributable to different criteria for positivity and the number of patients studied.

One might hypothesize that Fra-1 positivity in DCIS results from a growth response of tumour cells following transformation, and is therefore a consequence of malignancy. No evidence of an association between Fra-1 expression and prognostic factors in DCIS was found, implying that a high frequency of Fra-1 expression may be associated with early events in carcinogenesis. In accordance with this hypothesis, Chiappetta *et al.*^14^ showed that Fra-1 expression started to become detectable in fibroadenomas and in breast ductal hyperplasia. We confirmed their findings,^14^ as we did not detect Fra-1 staining in normal breast tissues used as controls.

We also observed a low frequency of HER-2 expression in invasive disease as compared to the DCIS cases, confirming previous reports.^22^–^26^ Like that of HER-2, Fra-1 expression may be characteristic of tumours at a discrete stage of pathogenesis. The studies of Latta *et al.*^26^ and Pommier *et al.*^27^ suggested that HER-2-negative clones are enriched in the transition to the invasive state, and invasive abilities may have been developed in the absence of HER-2 expression. A similar mechanism might explain the less common expression of Fra-1 in IDC than in DCIS. However, experimental data obtained with cells in culture showed that several genes associated with EMT, such as those encoding metalloproteinases, SPARC, vimentin and β1-integrin, were closely related to Fra-1 expression.^4^,^7^,^10^,^11^,^28^,^29^ Fra-1 transcription and stabilization against proteasome-dependent degradation is regulated via mitogen-activated protein kinase–extracellular signal-regulated kinase (ERK1/2),^30^ and previous studies have supported a mechanism by which Fra-1 acts downstream of ERK, inducing EMT and regulating cell migration.^28^,^31^,^32^

A recent study suggested that up-regulation of Fra-1 in tumour-associated macrophages, which is dependent on the interaction between stroma and breast tumour cells, may have a pivotal role in tumour progression.^33^ The mechanism may be related to activation of signalling pathways releasing soluble factors that, in turn, lead to increased production of angiogenic factors and proteases by tumour cells. We report here the presence of Fra-1 in fibroblasts associated with breast cancer, which are considered to be activated fibroblasts. Thus, epithelial tumor cells might be influenced by fibroblast in a similar mechanism.

In IDC, we noted an association of Fra-1 positivity with poor prognostic characteristics, including ER negativity, poor differentiation, and overexpression of HER-2, suggesting an association with aggressive disease. A negative correlation between Fra-1-positivity and ER-positive status has been already observed in breast cancer cell lines and IDC cases.^5^,^7^,^10^,^12^,^34^ It could be that, because of the lack of ER, other factors form complexes with Fra-1, and that changes in AP-1 composition, activation or nuclear retention may induce the expression of genes involved in the establishment of an aggressive phenotype.^34^,^35^

Our data indicate a positive association between Fra-1 and HER-2 in IDC. In agreement with this, one of the subgroups with the highest percentage of Fra-1 positivity (38.5%) is characterized by negativity for ER and PR and the presence of HER-2 overexpression. Thus, there is a subset of patients in whom HER-2 expression may be regulated by a pathway involving Fra-1.

In the present study, a higher percentage of Fra-1 expression (34%) was found in basal-like carcinomas than in the subgroup of luminal tumours. In breast carcinoma, basal-like and HER-2-positive/ER-negative tumour subtypes have been described to be associated with high expression of the ERK1/2 pathway.^36^,^37^ The presence of Fra-1 in basal-like carcinomas was expected, as basal-like cultured cells such as MDA-MB 231, BT549, MDA 435S, MX1, BT20 and H5578T cells have been shown to pre-ferentially express Fra-1.^4^,^5^,^10^,^12^,^38^

In view of our results, we subsequently addressed the expression of Fra-1 as a prognostic marker in our cohort of invasive breast cancer patients. Conventional prognostic factors, such as nodal status, tumour size and histological grade, and classifying subsets of patients according to ER, PR and HER-2 immunostaining expression had prognostic value in our study. On the other hand, in spite of being related to high grade, ER negativity, the presence of HER-2 and basal phenotype, Fra-1 expression was not translated into prognostic significance in terms of survival.

## Abbreviations

AP-1: activator protein 1
CK: cytokeratin
DCIS: ductal carcinoma *in situ*
EMT: epithelial–mesenchymal transition
ER: oestrogen receptor
ERK: extracellular signal-regulated kinase
Fra-1: Fos-related antigen 1
HER-2: human epidermal growth factor receptor 2
IDC: invasive ductal carcinoma
PR: progesterone receptor
TMA: tissue microarray
